# Room temperature sensing of CO_2_ using *C*3-symmetry pyridinium-based porous ionic polymers with triazine or benzene cores†

**DOI:** 10.1039/d4ra07062c

**Published:** 2025-02-03

**Authors:** Maha A. Alshubramy, Khalid A. Alamry, Hajar S. Alorfi, Sameh H. Ismail, Nadjet Rezki, Mohamed Reda Aouad, Salsabeel Al-Sodies, Mahmoud A. Hussein

**Affiliations:** a Chemistry Department, Faculty of Science, King Abdulaziz University Jeddah 21589 Saudi Arabia maha.alshubramy@gmail.com malshubramy@stu.kau.edu.sa maabdo@kau.edu.sa; b Egypt Nanotechnology Center, Cairo University El–Sheikh Zayed, 6th October Giza Egypt; c Department of Chemistry, Taibah University 30002 Al-Madina Al-Mounawara Saudi Arabia; d Chemistry Department, Faculty of Science, Assiut University Assiut 71516 Egypt mahussein74@yahoo.com

## Abstract

A new class of ionic polymers tethering triazine (benzene) core hybrids with three dipyridinium as cationic counterparts combined with bromide and/or chloride anions PPyBz-O_Br_ and PPyTri-O_Cl_ were successfully prepared *via* the alkylation of 4,4′-dipyridyl derivatives 4,4′-bp-O with 1,3,5-tris(bromomethyl)benzene BB and/or cyanuric chloride CC. The precursor, 4,4′-bp-O,was synthesized through the condensation of 4-pyridine carboxaldehyde and 4,4′-oxydianiline. The resulting ionic polymers, PPyBz-O_Br_ and PPyTri-O_Cl_, underwent metathetical anion exchange, forming new ionic polymers bearing LiTFSI and KPF_6_ as anions. Characterization of the synthesized hybrid molecules was performed through FTIR, ^1^H NMR, and ^13^C NMR analyses. PXRD and SEM showed semi-crystalline structures and a homogenous distribution of micro-/or nanoparticles. TGA and DTA displayed high thermal stability of the synthesized polymer. The sensing activity of the modified ionic polymers was examined using a quartz crystal nanobalance (QCN) for CO_2_ detection. The resulting sensor demonstrated the ability to provide precise, selective, and reproducible CO_2_ measurements.

## Abstract 2

A new class of *C*3-symmetry porous ionic polymers, tethering triazine (benzene) core hybrids, was successfully prepared and used for room-temperature CO_2_ sensing.

## Graphical abstract

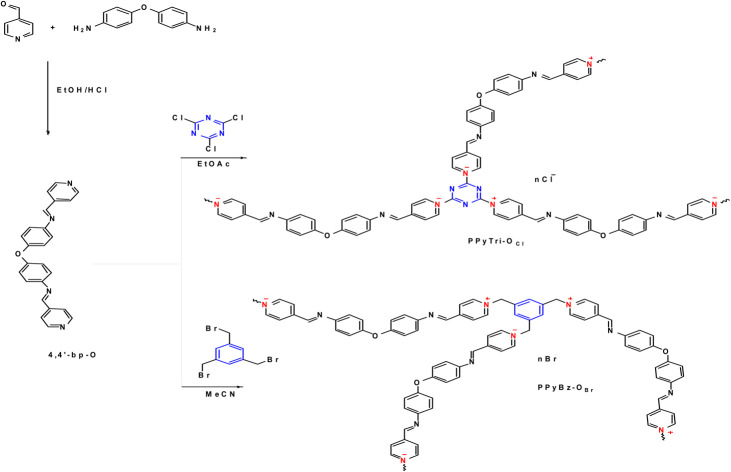

## 1. Introduction

The detection of carbon dioxide (CO_2_) is pivotal across diverse domains, including fire detection,^1^ greenhouse gas surveillance,^2^ air-quality monitoring,^3,4^ health,^5–7^ marine and environmental studies,^8^ and food supply chain management.^9,10^ Traditional CO_2_ sensing methods, such as gas chromatography,^11^ infrared spectrometry,^12^ fluorescence,^13–15^ photoacoustic spectroscopy,^16^ and severinghaus electrodes,^17^ offer high selectivity and sensitivity. However, they often suffer from drawbacks such as high costs, bulkiness, elevated power consumption, and susceptibility to electromagnetic interference.^16,18^ The development of highly sensitive, efficient, and cost-effective sensors is critical for on-site analysis and reducing CO_2_ emissions. The quartz crystal nanobalance (QCN) is a device that utilizes a quartz crystal microbalance (QCM) to detect mass changes at the nanogram level by measuring changes in the resonator frequency. By incorporating an appropriate chemical interface layer or recognition element, the QCN exhibits significant potential for CO_2_ sensing.^19^

CO_2_-responsive polymers have garnered significant attention due to their high flexibility and sensitivity at room temperature.^20–24^ These polymers undergo changes in physical attributes, such as electrical resistance, volume, and transparency, when exposed to CO_2_.^25^ Among the functional groups in these polymers, amidine,^26,27^ guanidine,^28^ and amines,^29,30^ especially tertiary amines, have been extensively studied for their CO_2_ responsiveness. Tertiary amines, with moderate base properties, exhibit good switchability between neutral and charged states. In contrast, primary and secondary amines form carbamate salts in the presence of CO_2_, making them less suitable for sensing applications.^31–33^

In recent years, considerable efforts have been made dedicated to the design, synthesis, and development of focused macromolecules with significant electrochemical features, which are the aims associated with various applications in organic synthesis. A wide range of porous materials such as covalent organic frameworks (COFs),^34^ porous organic polymers (POPs),^35^ metal–organic frameworks (MOFs),^36^ organic–inorganic hybrid materials^37^*etc.*, are attractive scaffolds for designing and developing various applications such as gas storage,^38–41^ photovoltaics,^42^ conductivity,^43–45^ batteries,^46–50^ catalysis,^51–54^ optoelectronic devices,^55^ and electrochemical sensing.^56^

While various advanced porous materials such as zeolites,^57^ metal–organic frameworks (MOFs),^58^ ionic liquids,^59,60^ phenolic resins, activated carbons, and carbon composites^61^ have been explored, POPs, particularly those with porous frameworks containing C–N linkages, have emerged as promising candidates for CO_2_ sensing. The dipole–dipole interaction between CO_2_ and N atoms in these frameworks enhances their effectiveness.^62–64^ One of the effective methods for incorporating C–N functional groups is to synthesize triazine with the rigid-shaped structure and *C*3-symmetry, utilizing a simple synthetic method not only without the formation of byproducts but also without the need for any kind of catalyst. Moreover, the other N-rich functionalities, such as pyridine, amine, amide, carbazole, or porphyrin units, need to be designed and synthesized reasonably, which have been proven to be efficient functional materials for gas storage/separation, heterogeneous catalysis, and sensing.^65,66^

However, the performance of QCM sensors depends significantly on the properties of the coating material used. Previous studies have explored a range of materials, including acrylonitrile–styrene copolymers (AS), tetramethylammonium fluoride tetrahydrate (TMAF), and polyethyleneimine (PEI) coatings. For example, AS-coated QCM sensors showed sensitivity to CO_2_ but were also highly affected by humidity, limiting their specificity for CO_2_ in mixed-gas environments.^67^ Similarly, TMAF-coated QCM sensors demonstrated sensitivity to multiple gases such as SO_2_, NH_3_, and CO_2_, with limited selectivity for CO_2_.^68^ More recently, multilayered systems involving materials such as PEI and nitrogen-doped tungsten carbide (WC@NC) have shown promise in mixed-gas classification but are complex to fabricate and optimize.^69^ Despite these advances, challenges remain in achieving high sensitivity, selectivity, and thermal stability for CO_2_ detection using QCM-based sensors. In this context, the design of porous materials with tailored functionalities presents a promising approach. Porous ionic polymers (PIPs), particularly those combining nitrogen-rich frameworks and electrostatic interaction sites, offer significant potential for CO_2_ sensing. However, previous work in this area has been limited by the lack of synergistic design elements that enhance both adsorption and sensor performance. In this study, we report a new class of porous ionic polymers (PIPs) designed for CO_2_ sensing, incorporating triazine (benzene) cores and pyridinium cations with PF_6_ and TFSI anions. The unique combination of triazine and pyridinium units introduces several advantages. The triazine core provides a nitrogen-rich, semi-crystalline framework with high thermal stability, enabling robust performance in harsh conditions. Pyridinium cations introduce electrostatic interaction sites, increasing affinity for CO_2_ molecules while maintaining selectivity over NH_3_ and H_2_S. Furthermore, the incorporation of PF_6_ and TFSI anions influences the material's morphology and crystallinity, optimizing gas diffusion and adsorption.

## 2. Experimental

### 2.1. Synthesis of *N*,*N*′-(oxybis(4,1-phenylene))bis(1-(pyridin-4-yl)methanimine) 4,4′-bp-O

A mixture of 4-pyridine carboxaldehyde (2 mmol) and 4,4′-oxydianiline (1 mmol) was dissolved in ethanol (25 mL) with drops of conc. HCl as a catalyst. The reaction mixture was heated under reflux for 3 h. Then, the precipitate formed was filtered, washed thoroughly with ethanol, dried, and recrystallized from acetone to give the desired Schiff bases 4,4′-bp-O. It was obtained as white crystals in 90%, Mp = 250 °C. FTIR (*ν*, cm^−1^): 3016 (s, Ar–H stretch), 1622 (C=N, imine), 1596 (C=N, pyridine), 1460 (Ar C=C bending), 1082 (Ar C–N bending), 829 (Ar C=C bending). ^1^H NMR (400 MHz, DMSO-*d*_6_): *δ*_H_ = 7.14 (d, 4H, *J* = 5.48 Hz, Ar–H̲), 7.45 (d, 4H, *J* = 5.84 Hz, Ar–H̲), 7.86 (d, 4H, *J* = 3.96 Hz, Ar–H̲), 8.75 (s, 2H, H̲C=N), 8.76 (d, 4H, *J* = 3.96 Hz, Ar–H̲). ^13^C NMR (DMSO-*d*_6_, 200 MHz): *δ*_C_ = 158, 156, 150, 146, 143, 123, 122, 119.

### 2.2. Synthesis of dipyridinium cationic polymers-based triazine and/or benzene core PPyBz-O_Br_ and PPyTri-O_Cl_

To a solution of 4,4′-dipyridyl, 4,4′-bp-O (1.5 mmol) in acetonitrile (20 mL) was added dropwise a solution 1,3,5-tris(bromomethyl)benzene BB and/or cyanuric chloride CC in acetonitrile (20 mL) under stirring. The reaction mixture was then refluxed for 72 h. The precipitate formed was collected by filtration, washed with chloroform (3 × 15 mL) dried to the desired ionic polymers PPyBz-O_Br_ and PPyTri-O_Cl_.

#### 2.2.1 Characterization of polymer PPyBz-O_Br_

PPyBz-O_Br_ was obtained as a yellow powder in 95% yield, FTIR: *ν* [cm^−1^]: 3385 (pyridinium salt stretch), 3116 (alkenyl C–H stretch), 3016 (Ar C–H stretch), 2970 (Alkyl C–H stretch), 1636 (pyridinium salt ring vibration), 1460 (Ar C=C bending), 1160 (Ar C–N bending), 829 (Ar C–C bending). ^1^H NMR (850 MHz, DMSO): *δ* (ppm) 8.77 (s, 1H), 8.49 (m, 2H), 8.29 (m, 2H), 8.02 (s, 1H), 7.94–7.33 (m, 4H), 5.80 (m, 2H). ^13^C NMR (400 MHz, DMSO): *δ*(ppm) 160.21, 149.96, 147.92, 145.22, 144.47, 140.07, 134.23, 132.76, 130.71, 129.54, 126.76, 66.06.

#### 2.2.2 Characterization of polymer PPyTri-O_Cl_

PPyTri-O_Cl_ was obtained as a brown powder in 75% yield, FTIR: *ν* [cm^−1^]: 3423 (pyridinium salt stretch), 3116 (alkenyl C–H stretch), 3016 (Ar C–H stretch), 1636 (pyridinium salt ring vibration), 1593 (triazine C=N), 1384 (C–N bending) 1460 (Ar C=C bending), 1160 (Ar C–N bending), 829 (Ar C–C bending). ^1^H NMR (850 MHz, DMSO): *δ* (ppm) 8.81 (m, 2H), 8.59 (m, 2H), 8.34 (m, 1H), 8.25–8.01 (m, 2H), 7.62–7.28 (m, 2H). ^13^C NMR (400 MHz, DMSO): *δ* (ppm) 171.86, 166.97, 155.94, 152.24, 141.35, 138.27, 133.66, 122.06.

### 2.3. Metathesis synthesis of ionic polymers PPyBz-O_PF6_, PPyBz-O_TFSI_, PPyTri-O_PF6_ and PPyTri-O_TFSI_

To a solution of ionic polymers PPyBz-O_Br_ and PPyTri-O_Cl_ (1 mmol) in acetonitrile was added a solution of lithium bis(trifluoromethane)sulfonimide (LiTFSI) and/or potassium hexafluorophosphate (KPF_6_) (1.2 mmol) and the solution was refluxed for 24 h. The solid formed was collected by filtration, washed with deionized water and dried at 100 °C.

#### 2.3.1 Characterization of polymer PPyBz-O_PF6_

PPyBz-O_PF6_ was obtained as a yellowish-orange powder in 90% yield, FTIR: *ν* [cm^−1^]: 3423 (pyridinium salt stretch), 3116 (alkenyl C–H stretch), 3016 (Ar C–H stretch), 2970 (Alkyl C–H stretch), 1636 (pyridinium salt ring vibration), 1460 (s, Ar C=C bending), 1160 (Ar C–N bending), 829 (Ar C–C bending), 863 (PF6^−^). ^1^H NMR (850 MHz, DMSO): *δ* (ppm) 8.85 (s, 1H), 8.59 (m, 2H), 8.41 (m, 2H), 8.15 (s, 1H), 8.03–7.37 (m, 4H), 5.92 (m, 2H). ^13^C NMR (400 MHz, DMSO): *δ*(ppm) 160.21, 148.24, 147.71, 145.16, 144.92, 140.31, 134.28, 132.98, 130.84, 129.83, 128.49, 126.87, 67.06. ^31^P NMR (162 MHz, DMSO) *δ* −131.01 to −157.36 (sep). ^19^F NMR (377 MHz, DMSO) *δ* −69.18 (s), −71.07 (s).

#### 2.3.2 Characterization of polymer PPyBz-O_TFSI_

PPyBz-O_TFSI_ was obtained as a light orange powder in 90%, FTIR: *ν* [cm^−1^]: 3423 (pyridinium salt stretch), 3116 (alkenyl C–H stretch), 3016 (Ar C–H stretch), 2970 (alkyl C–H stretch), 1636 (pyridinium salt ring vibration), 1460 (Ar C=C bending), 1160 (Ar C–N bending), 829 (Ar C–C bending), 1207 (SO_2_, TFSI) and 1353 (CF_3_, TFSI). ^1^H NMR (850 MHz, DMSO): *δ* (ppm) 8.85 (s, 1H), 8.59 (m, 2H), 8.41 (m, 2H), 8.15 (s, 1H), 8.03–7.37 (m, 4H), 5.92 (m, 2H). ^13^C NMR (400 MHz, DMSO): *δ*(ppm) 160.05, 148.16, 147.81, 145.36, 144.64, 140.23, 134.16, 132.74, 130.03, 128.65, 126.76, 66.98. ^19^F NMR (377 MHz, DMSO): *δ*(ppm) −73.49 (s).

#### 2.3.3 Characterization of PPyTri-O_PF6_

PPyTri-O_PF6_ was obtained as a reddish-brown powder in 90% yield, FTIR: *ν* [cm^−1^]: 3423 (pyridinium salt stretch), 3116 (alkenyl C–H stretch), 3016 (Ar C–H stretch), 1636 (pyridinium salt ring vibration), 1593 (triazine C=N), 1384 (C–N^+^ bending)1460 (Ar C=C bending), 1160 (Ar C–N bending), 829 (Ar C–C bending), 863 (PF6^−^). ^1^H NMR (850 MHz, DMSO): *δ* (ppm) 8.87 (m, 2H), 8.67 (m, 2H), 8.39 (s, 1H), 8.33–8.19 (m, 2H), 7.73–7.38 (m, 2H). ^13^C NMR (400 MHz, DMSO): *δ* (ppm) 171.79, 167.11 154.94, 152.24, 141.18, 138.41, 133.76, 121.99. ^31^P NMR (162 MHz, DMSO) *δ* −131.01 to −157.36 (sep). ^19^F NMR (377 MHz, DMSO) *δ* −69.17 (s), −71.06 (s).

#### 2.3.4 Characterization of polymer PPyTri-O_TFSI_

PPyTri-O_TFSI_ obtained as a brown powder in 95% yield, FTIR: *ν* [cm^−1^]: 3423 (s, pyridinium salt stretch), 3116 (w, alkenyl C–H stretch), 3016 (s, Ar C–H stretch), 1636 (*vs.*, pyridinium salt ring vibration), 1593 (s, triazine C=N), 1384 (s, C–N^+^ bending)1460 (s, Ar C=C bending), 1160 (s, Ar C–N bending), 829 (*vs.*, Ar C–C bending), 1207 (SO_2_, TFSI) and 1353 (CF_3_, TFSI). ^1^H NMR (850 MHz, DMSO): *δ* (ppm) 8.77 (m, 2H), 8.55 (m, 2H), 8.30 (s, 1H), 8.21–8.06 (m, 2H), 7.60–7.25 (m, 2H). ^13^C NMR (400 MHz, DMSO): *δ* (ppm) 171.93, 167.04 155.94, 152.31, 141.03, 138.41, 133.76, 122.06. ^19^F NMR (377 MHz, DMSO) *δ* −73.49 (s).

### 2.4. Fabrication of PPyTri-O and PPyBz-O CO_2_ sensors

The QCN was used to evaluate the PPyTri-O and PPyBz-O activity toward CO_2_ detection. In order to ensure a clean surface, the piranha solution (a blend of sulfuric acid and hydrogen peroxide) was used to clean the QCN chips, and the samples were rinsed with deionized water and dried using nitrogen. To create conductive pathways for the PIP sensors, the gold electrode was deposited on the QCN chip using a sputtering deposition method. The PIP layers were then applied using the spin coating technique and connected to the measurement system using bond wires. The CO_2_ chamber was prepared by installing inlets and outlets for gas flow and a temperature control system of 20 °C, 25 °C, and 30 °C using Peltier temperature baths. CO_2_ gas was then introduced into the chamber, and the sensor responses were analyzed to evaluate the performance of the PIP sensors under different temperature conditions.

## 3. Results and discussion

### 3.1. Synthesis and characterization

**Scheme 1:**
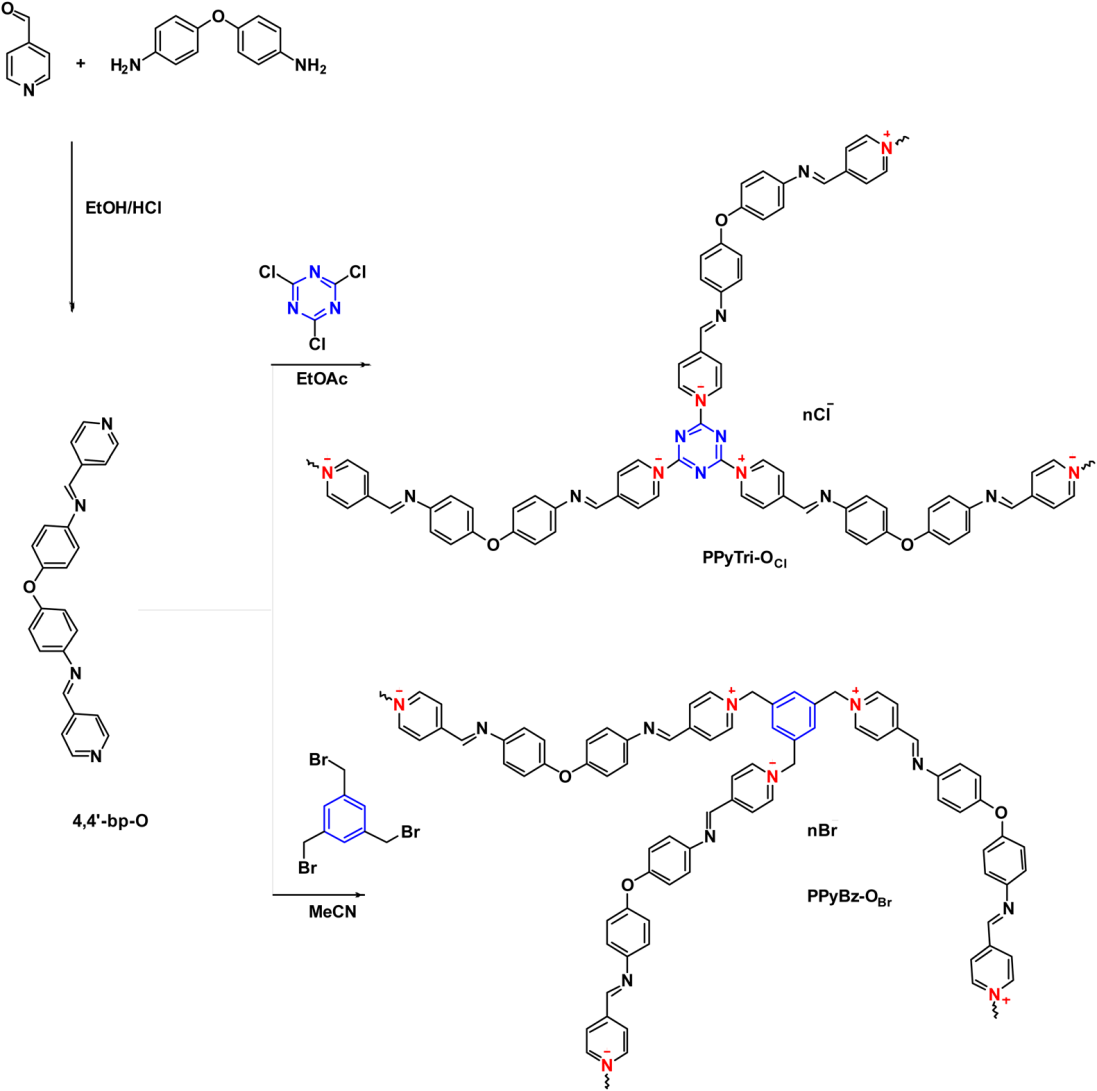
Synthesis of the precursor Schiff base 4,4′-bp-O and targeted *C*3-symmetry porous ionic polymers PPyBz-O_Br_, PPyTri-O_Cl_.

**Scheme 2:**
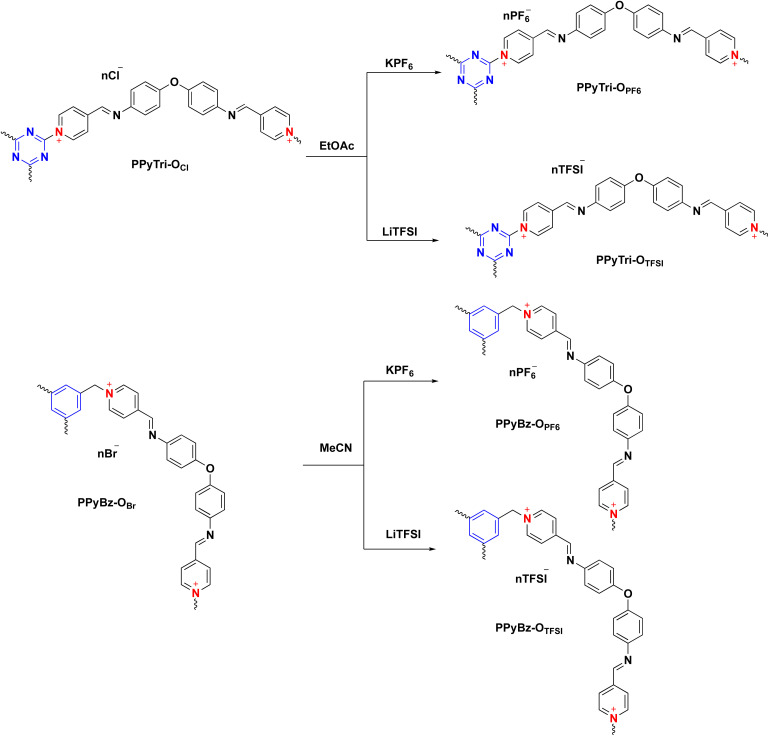
Metathesis of *C*3-symmetry porous ionic polymers.

The design of *C*3-symmetry porous ionic polymers (PIPs) involved careful consideration of the core structure and linkers. Thus, in the present work, new ionic polymers with a *C*3-symmetry structure were synthesized and investigated for their CO_2_ detection. The benzene and/or triazine core, viologen as linkers, and PF_6_^−^ and TFSI^−^ as counter anions were combined in the same framework of the tailored polymers in order to improve the electrostatic and aromatic properties impacting their overall behavior as well as CO_2_ sensing. The synthesis of the targeted *C*3-symmetry pyridinium ionic polymers PPyBz-O_Br_, PPyBz-O_PF6_, PPyBz-O_TFSI_, PPyTri-O_Cl_, PPyTri-O_PF6_, and PPyTri-O_TFSI_ was accomplished using the synthetic methodology depicted in Schemes 1 and 2. The first step involved the synthesis of the precursor's Schiff base 4,4′-bp-O through thermal condensation of the 4-pyridine carboxaldehyde with 4,4′-oxydianiline in refluxing ethanol and in the presence of the catalytic amount of acid. The ^1^H NMR spectra of the monomer 4,4′-bp-O showed characteristic signals at 7.14–8.76 ppm and 7.09–8.76 ppm attributed to the aromatic protons of the phenyl and pyridine rings, respectively. While the imine proton (HC=N) appeared as a distinct singlet at 8.75 ppm.

**Fig. 1:**
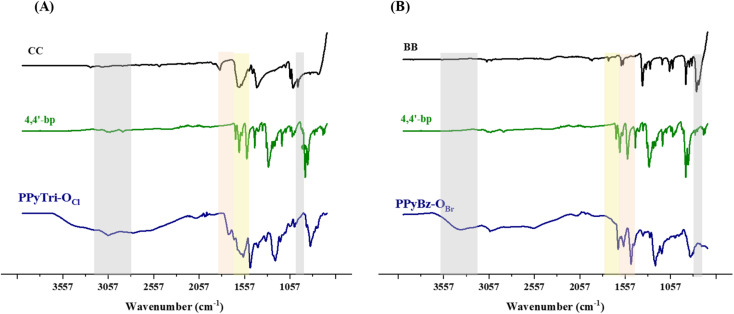
FTIR spectra of the precursor Schiff base 4,4′-bp-O and targeted *C*3-symmetry porous ionic polymers. (A) Triazine core and (B) benzene core.

The Menshutkin reaction was adopted as a common method for generating polymers-based quaternary pyridinium as ammonium salt. Thus, the appropriate 1,3,5-tris(bromomethyl)benzene BB and/or cyanuric chloride CC were used in the polymerization process through their conjunction with the synthesized bis(1-(pyridin-4-yl)methanimine) Schiff base 4,4′-bp-O as linkers to furnish the desired cationic polymers PPyBz-O_Br_ and PPyTri-O_Cl_ bearing chloride and bromide as counter anions, as elucidated in Scheme 1. The success of the synthesis of the targeted *C*3-symmetry ionic polymers was supported by the investigation of their spectral data based on FTIR and NMR results. Thus, the FTIR spectrum of the polymer PPyTri-O_Cl_ (Fig. 1(A)) clearly showed the disappearance of the stretching vibration near 850 cm^−1^ attributed to the C–Cl band of the starting material CC, supporting the quaternization of pyridine ring in the monomer 4,4′-bp-O. A new intense band was observed near 1645 cm^−1^ belonging to pyridinium cations, which is another piece of evidence for the success of the quaternization reaction and formation of the targeted ionic polymers PPyTri-O_Cl_. The spectra also showed a shift of the C=N absorption bands of the triazine ring (1508 cm^−1^) compared to their precursor CC. The C=N absorption of the azomethine linkage and pyridine ring were observed at 1630 and 1650 cm^−1^, respectively. Similarly, no stretching bands near 690 cm^−1^ were recorded in the FTIR spectra of the polymers tethering to the benzene core PPyBz-O_Br_ (Fig. 1(B)), confirming the absence of the C–Br bond in their structure and confirming their involvement in the alkylation of the pyridine nucleus yielding the desired adduct PPyBz-O_Br_, with the quaternary nitrogen atoms (N^+^) exhibiting an intense band around 1645 cm^−1^, evidencing the proposed structures. In addition, the band observed at 1640 cm^−1^ confirmed the presence of the imine linkage in the framework of the resulting PPyBz-O_Br_ ionic polymer.

**Fig. 2:**
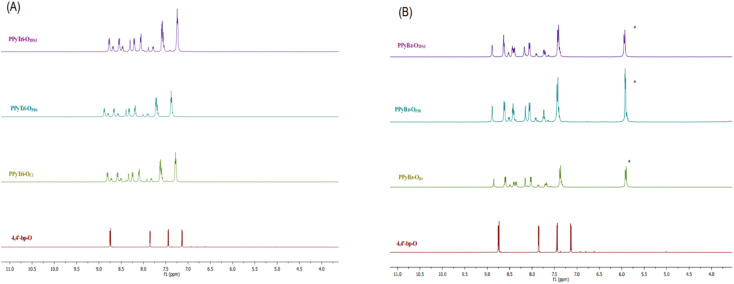
^1^H NMR spectra of the precursor Schiff base 4,4′-bp-O and the targeted *C*3-symmetry porous ionic polymers. (A) Triazine core and (B) benzene core.

On the other hand, the structures of the synthesized polymers PPyBz-O_Br_, PPyBz-O_PF6_, PPyBz-O_TFSI_, PPyTri-O_Cl_, PPyTri-O_PF6_, and PPyTri-O_TFSI_ were elucidated based on their NMR spectra. The ^1^H NMR spectra of the developed polymers-based triazine core PPyTri-O_Cl_ and their analogs PPyTri-O_PF6_ and PPyTri-O_TFSI_ (Fig. 2(A)) exhibited a broad signal in the aromatic area ranging from 7.25 to 8.87 ppm due to the polymeric backbone effect. The imine protons resonated around 8.30–8.39 ppm. Moreover, the carbons of the triazine ring and imine were recorded at 171.86–171.93 ppm and 166.97–167.04 ppm, respectively, in their ^13^C NMR spectra. The remaining aromatic carbons were observed in their appropriate area (121.80 to 155.94 ppm) and are detailed in the experimental section.

The ^1^H NMR of the polymers comprising the benzene nucleus, PPyBz-O_Br_, PPyBz-O_PF6_, and PPyBz-O_TFSI_ (Fig. 2(B)), revealed additional signals at 5.80 to 5.94 ppm attributed to methylene protons (CH_2_), confirming the incorporation of the benzene core in the polymer structures resulting from the quaternization reaction. Their NMR spectra revealed the same broadness as appeared on the first set of the triazine-ionic polymers. The spectra also displayed characteristic singlets at 8.02, 8.19, and 8.15 ppm belonging to the imine protons (CH=N) with the same shift impact that seemed in the triazine core results. Extra aromatic protons resonated at 8.77 and 8.85 ppm confirming the presence of the benzene rings. The ^13^C NMR spectra showed new signals ranging from 66.06 to 66.98 ppm, revealing the presence of the methylene carbons (CH_2_).

The resulting cationic polymers, PPyBz-O_Br_ and PPyTri-O_Cl_, which incorporate chloride (Cl^−^) and bromide (Br^−^) as counter anions, undergo a metathesis reaction. This anion exchange process occurs when the polymers are treated with a saturated brine solution of LiTFSI and KPF_6_, yielding the cationic polymers with PF_6_^−^ and TFSI^−^ as counter anions: PPyBz-O_PF6_, PPyBz-O_TFSI_, PPyTri-O_PF6_ and PPyTri-O_TFSI_. The success of the metathesis reaction was confirmed and supported by the investigation of the spectral data of the resulting polymers PPyBz-O_PF6_, PPyBz-O_TFSI_, PPyTri-O_PF6_ and PPyTri-O_TFSI_ tethering the PF_6_ and TFSI as counter anions. Their FTIR spectra (Fig. S1†) showed the appearance of the absorption bands at 863 cm^−1^, 1207 cm^−1^, and 1353 cm^−1^, confirming the presence of PF6^−^, SO_2_, and CF_3_, respectively, of TFSI^−^ anions. The analysis of ^19^F NMR and ^31^P NMR (Fig. S2†) of the polymers PPyBz-O_PF6_, PPyBz-O_TFSI_, PPyTri-O_PF6_, and PPyTri-O_TFSI_ were in accordance with their structures. The polymers PPyBz-O_PF6_ and PPyTri-O_PF6_ displayed distinguished two singlets at −69.17 and −71.06 ppm and −69.18 and −71.07 ppm, respectively. In addition, the PF_6_^−^ anion exchange was also confirmed from the resonance of distinct septet in the range of −131.01 to −157.36 ppm appearing in the ^31^P NMR spectra of PPyBz-O_PF6_ and PPyTri-O_PF6_ polymers. The TFSI^−^ anions appeared as a singlet near −73.49 ppm in the ^19^F NMR spectra of PPyBz-O_TFSI_ and PPyTri-O_TFSI_ polymers supporting their structures (Fig. S2†).

### 3.2. Morphology study

**Fig. 3:**
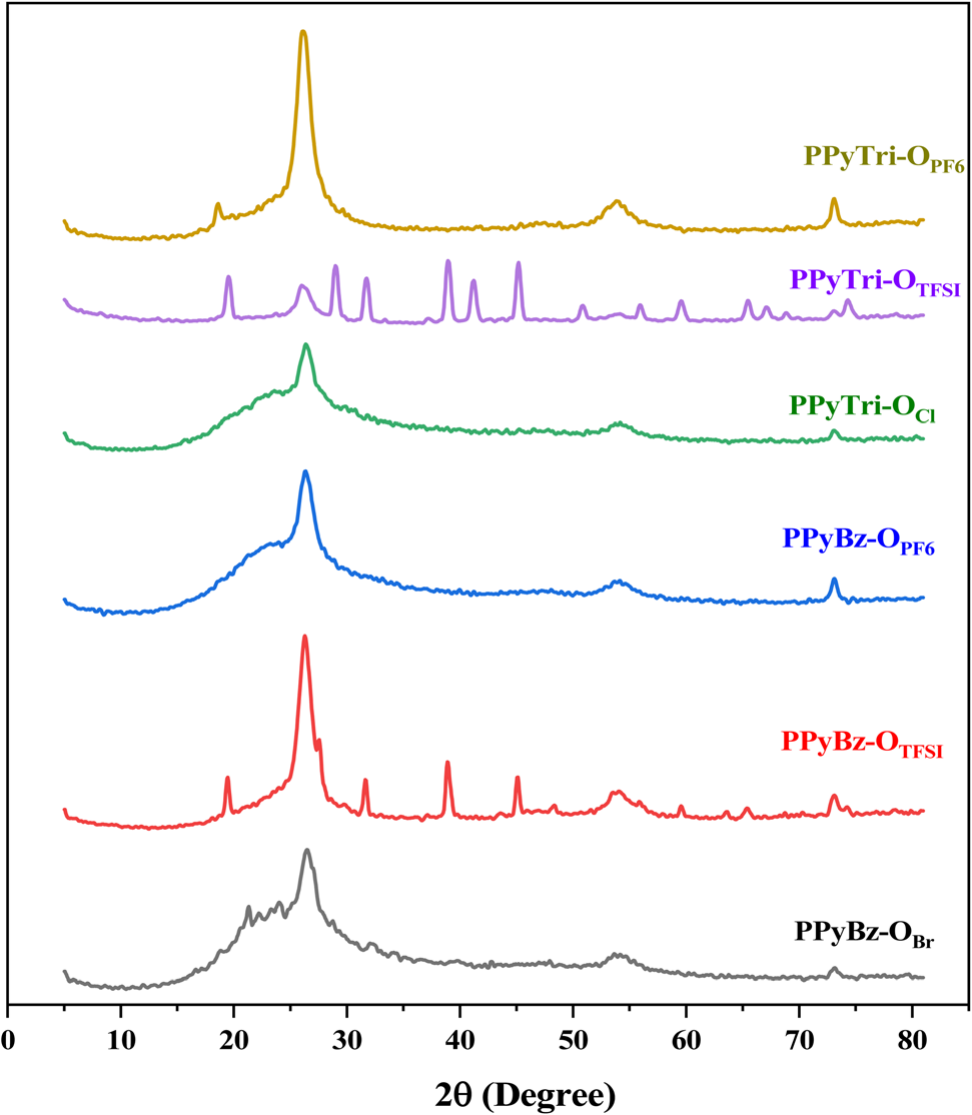
PXRD patterns of the synthesized *C*3-symmetry porous ionic polymers.

Powder X-ray diffraction (PXRD) was utilized to analyze the crystalline nature of the synthesized polymers (Fig. 3). The PXRD patterns showed semi-crystalline behavior for all formed polymers, with four main characteristic peaks at 2*θ* = 19.3°, 26.4°, 54.4°, and 72.9°. The diffraction peaks at 2*θ* = 10°–30° and 2*θ* = 35°–50° were the confirmation of the (002) and (101) planes of the carbon materials, respectively. The peak at 10°–30° indicated the fragmented and devolved crystallite aromatic structure, while a broad and weak peak at 35°–50° indicated an amorphous structure with minimal graphitization degree.^70^

The exchange anions affected the crystallinity of the designed two core polymers due to the flexible structure *via* the imine and aliphatic bridges between the rigid aromatic rings. The anions displacement by the TFSI anion in PPyBz-O_TFSI_ and PPyTri-O_TFSI_ polymers caused an increase in the crystallinity in the PXRD patterns and showed diffraction peaks at 19.5°, 28.9°, 31.7°, 39.0°, 45.1° that matched (201), (114), (015), (116), (125) crystal planes, respectively, and were consistent with the data from the JCPDS card (no: 01-082-2341) of TFSI. The bulky size of the TFSI ion affected the natural crystal packing due to the formation of the bilayer structure with no significant change in the crystallinity of PF_6_. This structural flexibility has also been reported in porous materials.^71–73^

**Fig. 4:**
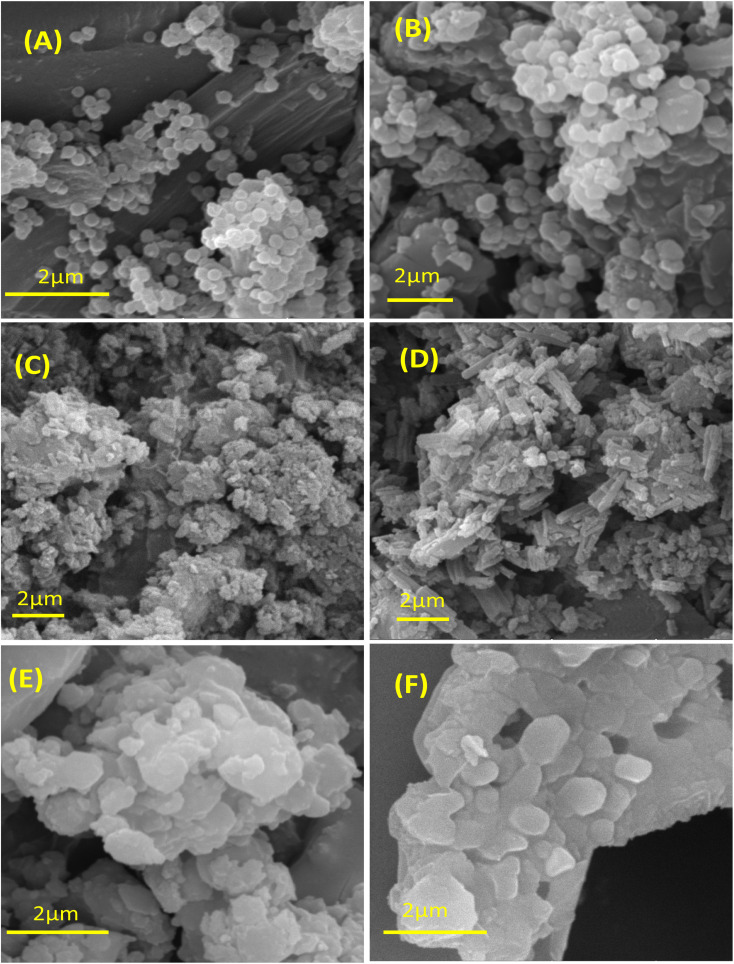
SEM images of the synthesized porous ionic polymers with *C*3-symmetry (A) PPyBz-O_Br_, (B) PPyTri-O_Cl_, (C) PPyBz-O_PF6_, (D) PPyTri-O_PF6_, (E) PPyBz-O_TFSI_ and (F) PPyTri-O_TFSI_.

To obtain in-depth insight into the impact of the anions on the morphology of the *C*3-symmetry porous ionic polymers, the synthesized polymers were examined using scanning electron microscopy (SEM) at a magnification of 20 k*x*, as shown in (Fig. 4(A–F)). In the case of the polymers PPyTri-O and PPyBz-O, distinct and well-defined morphologies were observed, featuring sphere-shaped structures with sizes ranging from nanosized. Conversely, both PPyTri-O_PF6_ and PPyBz-O_PF6_ exhibited aggregated clusters, coarse surfaces, and loosely arranged irregular particles. Similarly, the polymers PPyTri-O_TFSI_ and PPyBz-O_TFSI_ showed aggregated and irregular particles in the micron-scale dimensions. These SEM images vividly depict the significant impact of bulky anions on the polymer morphology. This observation aligns with findings reported by E. Maya *et al.*^74^

### 3.3. Thermal study

The thermal properties of the synthesized *C*3-symmetry ionic polymers were studied using thermogravimetric analysis (TGA) and derivative thermogravimetry (DTG) in the temperature range of 25–1000 °C at a heating rate of 10 °C min^−1^ (Fig. S3†). The TGA/DTG curves exhibited three-step weight loss. Most synthesized polymers were not hygroscopic in nature, and the first stage of weight loss occurred between 200 °C and 600 °C, while the second stage was observed between 630 °C and 840 °C owing to the degradation of the polymer backbone.^70,75–77^

Table 1 presents a comprehensive comparison of *T*_10_, *T*_25_, and *T*_50_, illustrating thermal decomposition at 10%, 25%, and 50%, respectively, for the polymers. The values of *T*_10_, *T*_25_, and *T*_50_ in the case of triazine core, the polymer with PF6 anion has the highest thermal stability, while in the case of benzene core, the anion with TFSI has the highest thermal stability. Moreover, Table 1 outlines the final temperature for the polymer degradation (PDT_final_) and the maximum polymer decomposition temperature (PDT_max_). As exemplified by Table 1, the PDT_final_ values ranged from 680 °C to 830 °C, while the DTG revealed PDT_max_ in the range of 670–800 °C. The bulky anion TFSI with triazine core shows the highest lowest values of PDT_final_ and PDT_max_ in comparison with the other anions. While the polymers with PF6 anion have the same value of PDT_final_ in two cores; however, the PDTmax value shows the highest thermal stability in the triazine core. From the polymers under investigation, it was found that the triazine core had more thermal stability, which may be due to less flexibility, and the bulky anion TFSI also increases the thermal stability by increasing the electrostatic effect.

**Table 1:** Thermal behavior of the synthesized *C*3-symmetry porous ionic polymers

	*T*_10%_	*T*_25%_	*T*_50%_	*T*_final_	*T*_max_
PPyTri-O_Cl_	280	320	380	740	690
PPyTri-O_PF6_	270	370	470	680	700
PPyTri-O_TFSI_	220	360	400	830	800
PPyBz-O_Br_	230	275	360	765	730
PPyBz-O_PF6_	160	190	300	685	670
PPyBz-O_TFSI_	300	370	460	770	720

### 3.4. Nitrogen adsorption analysis

Nitrogen adsorption–desorption isotherms, as shown in Fig. S4,† were taken to characterize the pores in the *C*3-symmetry ionic polymer. All the polymers exhibited type IV nitrogen adsorption–desorption isotherms.^78^ The adsorption equilibrium leads to the growth of the H3 hysteresis loop, associated with capillary condensation in mesoporous structures, including the accumulation of the slit pore formation.^79^Table 2 shows the specific surface area calculated using the Bruner–Emmett–Teller (BET) theory model and density functional theory (DFT), as well as pore volume and average pore size calculated using the BJH model and DFT. Accordingly, the pore-size distributions were evaluated through BJH and DFT, approved mesopores character (more than 2 nm pores) for all the pore-size distribution patterns (Fig. S4†). Table 2 shows that the polymers with the triazine core have a higher specific surface area when compared with polymers with benzene cores for the same anion. That may be due to the flexibility of methylene in the benzene core that reduces the surface area.^80^ However, when the anion is exchanged by PF_6_ or TFSI for the same core, it is found that TFSI has a lower surface area compared to PF_6_. The same pattern is found in the benzene and triazine cores, and that may affect the bulky anions to reduce the surface area. Notably, the counter anions occupy some spaces within the networks of these porous polymers, more or less leading to reduced surface areas.^81^ On the other hand, for the ionic porous polymers with *C*3-symmetry, the rigidity of skeletons was more or less weakened by the tritopic building blocks comprising phenyl and/or triazine rings, which could create much more complex conformational torsions by free rotation around single carbon–carbon bonds, and also tends to cause the interpenetration of chain segments. Correspondingly, these three porous polymers show lower specific surface areas.^82,83^

**Table 2:** Surface area, pore volume, and pore size for synthesized *C*3-symmetry ionic polymers

Sample code	Surface area (m^2^ g^−1^)			Pore volume (cm^3^ g^−1^)		Pore size (nm)	
	BET	BJH	DFT	BJH	DFT	BJH	DFT
PPyTri-O_Cl_	54.73	58.40	52.83	0.17	0.17	5.7	6.9
PPyTri-O_PF6_	60.57	64.66	57.73	0.18	0.18	5.7	6.3
PPyTri-O_TFSI_	35.00	26.76	33.78	0.11	0.10	5.7	6.3
PPyBz-O_Br_	50.76	34.18	54.49	0.19	0.19	5.7	6.3
PPyBz-O_PF6_	53.84	56.30	56.61	0.21	0.19	5.7	6.3
PPyBz-O_TFSI_	46.15	37.60	46.35	0.15	0.15	5.7	6.3

### 3.5. QCN study

#### 3.5.1 Sensor activity of PPyTri-O and PPyBz-O derivatives on QCN chips for CO_2_ gas sensing

**Fig. 5:**
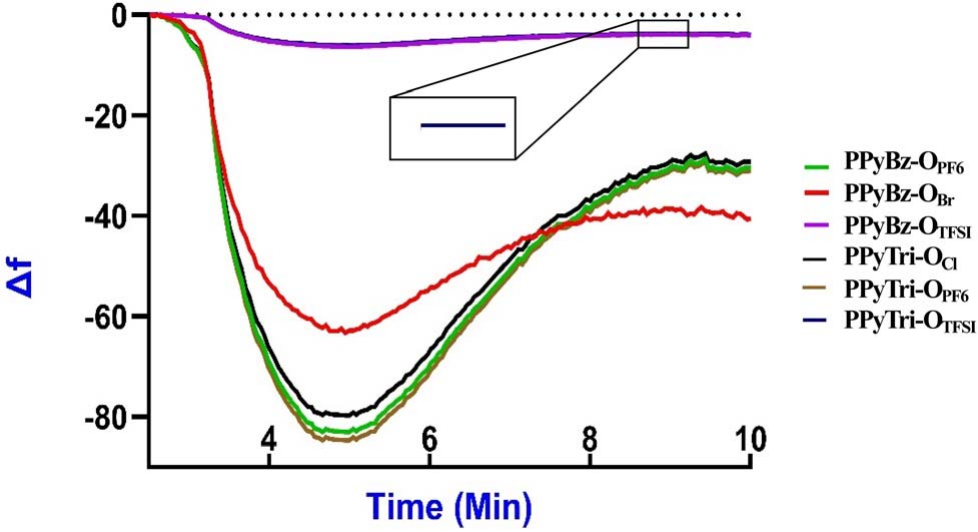
QCN sensorgram curves of PPyTri-O and PPyBz-O thin film derivatives showing the response to CO_2_ gas at 25 °C.

The detection of CO_2_ on the surface of porous ionic polymers (PIPs) sensors was performed and measured using QCN. A comparative frequency shift (Δ*f*) analysis of CO_2_ gas exposure across PPyTri-O and PPyBz-O derivatives films at 20 °C, 25 °C, and 30 °C was performed. The process was conducted sequentially at each temperature, providing sufficient time for the QCN chips to stabilize after each temperature adjustment, and the baseline frequency (*f*0) for each PIP sensor was recorded before CO_2_ gas exposure.^84–86^Fig. 5 illustrates the monitoring of the frequency shift (Δ*f*) for each individual PIPs sensor while the CO_2_ gas is introduced into the chamber. The (Δ*f*) values and the average of (Δ*f*) were recorded for every CO_2_ concentration level ranging from low to hundreds of ppm. Upon the introduction of CO_2_ gas to the sensors, it was observed that the frequency decreased rapidly, and the response time of the PPyTri-O and PPyBz-O derivatives was established as the time required for the frequency shift to reduce by ∼85% upon activation of the gas. The polymers PPyTri-O_PF6_ and PPyBz-O_PF6_ displayed the highest adsorption of CO_2_ on their surfaces, followed by PPyTri-O_Cl_ and PPyBz-O_Br_, while the PPyBz-O_TFSI_ and PPyTri-O_TFSI_ recorded the least response. The response time for the four polymers was between 4 to 5 min. As the gas molecules were adsorbed onto the surface of the sensitive film, the sensors reached a steady state. When the CO_2_ gas was purged from the sensors using clean N_2_, the frequency returned to its original level, and the sensors demonstrated quick recovery with approximately 2 min. These data demonstrated that the type of counter anion significantly influences the CO_2_ adsorption capacity of PIPs. Among the four polymers, the ionic polymers with PF_6_ as the counter anion attained the best results, followed closely by PIPs containing halogens and those with TFSI were the last. These results were consistent with the surface area findings derived from the BET model theory, where the PIPs displayed a surface area in the same order as their response to CO_2_ adsorption, PF_6_ >Cl, Br > TFSI. The obtained results are in agreement with those from the prior research on polymerization-derived PIPs,^81,82^ which indicated that inorganic anions such as halogens and PF_6_ provided the most effective CO_2_ adsorption. This concludes that selecting the right anion is crucial for achieving high CO_2_ detection performance and increasing the CO_2_ adsorption on the surface of the formed sensor. Furthermore, the influence of the cations on the CO_2_ detection comes right after the anions impact and can be observed in the difference responses between PIPs with triazine and benzene cores in PPyTri-O_PF6_ and PPyBz-O_PF6_ as well as in PPyTri-O_Cl_ and PPyBz-O_Br_. The ionic polymers with triazine entities showed a higher affinity for CO_2_ than those with a benzene core, which can be predicted by the effect of C–N linkages on attractive CO_2_ adsorption.^87^

#### 3.5.2 Precision analysis

**Fig. 6:**
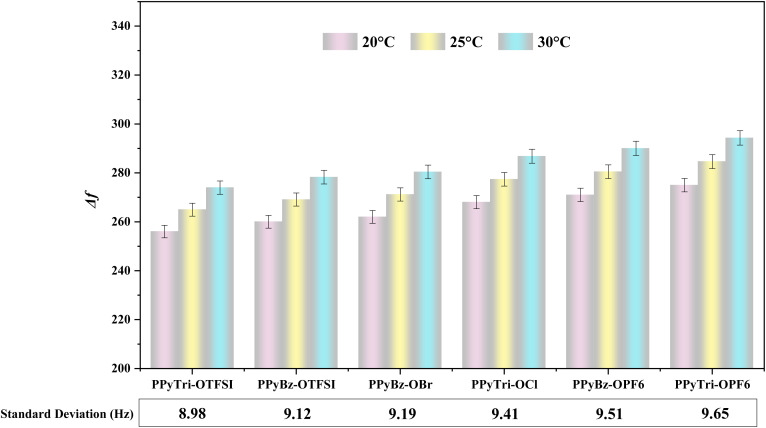
The average frequency shift (Δ*f*) and standard deviation for each PIP sensor at 20 °C, 25 °C, and 30 °C.

**Fig. 7:**
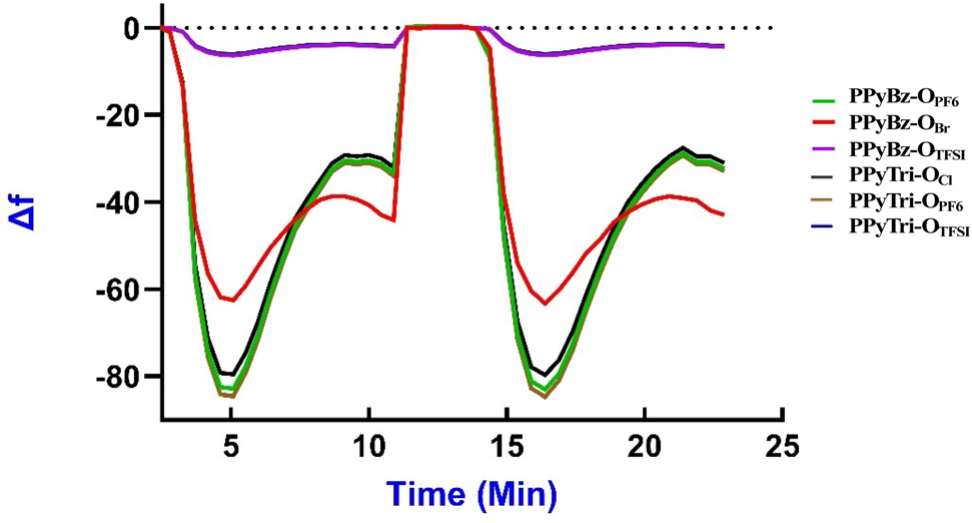
Sensorgram curves of PIPs after 25 minutes of exposure showing a high level of precision of the sensors.

The precision of the PIP sensors was assessed by calculating the standard deviations of the Δ*f* values after 25 minutes of gas exposure for the PIP sensors on the QCN chips (Fig. 6). The analysis confirmed that the mean frequency shifts of the sensors were consistent with 25 minutes CO_2_ gas exposure experiments at different temperatures (Fig. 7). Fig. 6 and 7 depict the mean Δ*f* values for the sensors, and the standard deviation of the repeated exposures ranged from 8.98 to 9.65 Hz, indicating a high level of precision and repeatability. These results demonstrate the exceptional precision and reliability of the PIP sensors for CO_2_ gas detection at temperatures between 20 °C and 30 °C. According to the data in Fig. 6, it can be observed that the impact of the temperature on the frequency shift becomes more pronounced as the temperature rises from 20 °C to 30 °C.

#### 3.5.3 Selectivity, reproducibility, and sensitivity

The selectivity of the fabricated PIP sensors on QCN chips for CO_2_ gas sensing at varying temperatures was studied in comparison to non-targeted gases such as NH_3_ and H_2_S (Fig. 6 and S5†). The obtained sensors outperformed the selectivity toward CO_2_ gas in comparison with NH_3_ and H_2_S as the Δ*f* values decreased, indicating CO_2_ gas adsorption on the surface. In contrast, the decrease in Δ*f* values for NH_3_ and H_2_S indicates the non-sensing of these gases (Fig. S5†). These findings demonstrate the exceptional CO_2_ selectivity of the sensors at different temperatures, making them a suitable choice for CO_2_ detection applications.

**Fig. 8:**
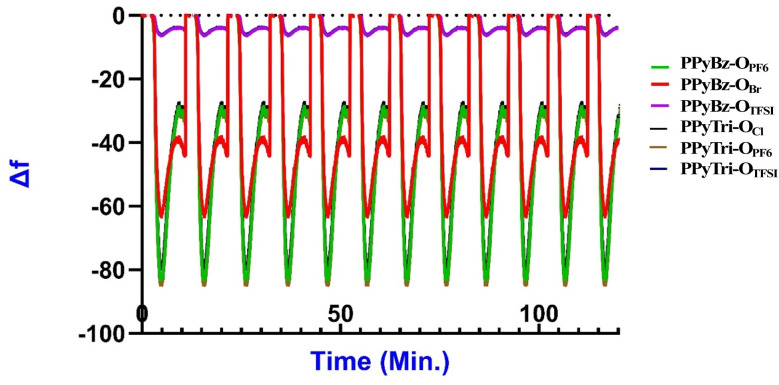
The repeatability response sensorgram curves of PIPs for multiple cycles.

The reproducibility test was undertaken through multiple repeated measurements of the frequency shift (Δ*f*) for randomly chosen PIPs at three temperatures (20 °C, 25 °C, and 30 °C). The relative standard deviations (RSD) of the Δ*f* values were computed to evaluate the sensors' reproducibility. Table S1† and Fig. (8) exhibit the frequency shift measurements (Δ*f*) and RSD (%) values for the samples, respectively. The RSD values in the table indicate that the frequency shifts between the triplicate measurements demonstrate a high level of reproducibility, and the outcomes in the figures for each run were stable and indistinguishable, as there were no noticeable differences in the response. These results demonstrate the dependability of the designed sensors when exposed to CO_2_ gas under various temperature conditions.

Evaluating the efficiency of the formed sensors on the QCN chips as CO_2_ gas sensors at various temperatures provides vital information regarding the capabilities of the sensors. Sensitivity can be represented as the change in the frequency (Δ*f*) per unit concentration of CO_2_ and can be calculated by dividing the average frequency shift (Δ*f*) by CO_2_ concentration.

#### 3.5.4 Mechanism of action

The fabricated PIP sensors on quartz crystal nanobalance (QCN) chips detected CO_2_ gas by leveraging the CO_2_ affinity of Lewis acidic groups within the PIPs. When CO_2_ molecules permeate the sensor phase, the polymer chains undergo structural changes, increasing rigidity. This rigidification due to CO_2_ adsorption causes a steady frequency shift. The primary transduction mechanism involves depositing polymer film layers containing CO_2_-philic moieties (*e.g.*, PF_6_) onto the piezoelectric quartz crystal surface. When CO_2_ gas interacts with the PIP sensor, the polar moieties of the porous ionic polymer form host–guest complexes with CO_2_ molecules, increasing the overall mass and reducing conformational chain flexibility, which stiffens the PIP film. Consequently, the frequency shift (Δ*f*) is directly proportional to mass change (Δ*m*) and inversely proportional to the square of the base resonance frequency (*f*0), as described by the Sauerbrey equation:Δ*f* = −(2·*f*0^2^·Δ*m*)/(*A*·*ρ*·√*μ*)where *A* is the electrode area, *ρ* is the density of quartz, and *μ* is the shear modulus.

The designed PIP sensors on QCN chips enable precise, selective, and reproducible CO_2_ measurements due to the stability of the physisorption process. This process forms weak intermolecular bonds between the CO_2_ molecules and the PIP-coated QCN chip surface, ensuring stable and continuous linkages during gas adsorption and desorption.

To contextualize the performance of the synthesized porous ionic polymers for CO_2_ sensing, a comparison was made with the previously reported QCM-based sensing materials. Table 3 summarizes the frequency shifts observed for CO_2_ detection using various materials, alongside their selectivity toward interfering gases such as NH_3_ and H_2_S. Ethyl cellulose (EC) exhibited strong CO_2_ responsiveness, while polypyrrole (PPy) demonstrated limited sensitivity. Tetramethylammonium fluoride tetrahydrate (TMAF) and polyethyleneimine (PEI) showed substantial CO_2_ responses; however, their cross-sensitivity to NH_3_ and SO_2_ limits their practical applicability. Similarly, nitrogen-doped tungsten carbide (WC@NC) provided excellent selectivity for H_2_S but showed minimal sensitivity to CO_2_. In comparison, the newly developed porous ionic polymers (*e.g.*, PPyTri-O_TFSI_, PPyBz-O_TFSI_, and PPyTri-O_PF6_) exhibited high CO_2_ sensitivity with frequency shifts ranging from 264.96 Hz to 284.63 Hz, demonstrating superior performance compared to the previously reported materials. Furthermore, these polymers exhibited exceptional selectivity for CO_2_, with minimal interference from NH_3_ and H_2_S, which is critical for real-world applications. The tunable performance of these polymers, achieved through structural modifications and tailored anion incorporation, further underscores their potential as next-generation materials for precise and selective CO_2_ sensing.

**Table 3:** Sensing of CO_2_ and selectivity over H_2_S and NH_3_ in PIPs and some reported materials

Material	Frequency shift for CO_2_ (Hz)	Selectivity CO_2_ over H_2_S	Selectivity CO_2_ over NH_3_	Notes	Ref.
Ethyl cellulose (EC)	438.95	Moderate	Low	Strong CO_2_ response; limited cross-responsiveness	88
Polypyrrole (PPy)	59.00	Low	Low	Modest sensitivity to CO_2_, mainly used in gas mixture detection	
Tetramethylammonium fluoride (TMAF)	300.80	Moderate	Low	Good CO_2_ sensitivity but notable responses to SO_2_ and NH_3_	
Polyethyleneimine (PEI)	980.78	Low	Low	Exceptional response to NH_3_, making it less selective for CO_2_	
WC@NC (nitrogen-doped tungsten carbide)	11.78	Low	Low	Excellent H_2_S selectivity but minimal CO_2_ sensitivity	
PPyTri-O_TFSI_	264.96	High	High	High CO_2_ sensitivity with excellent selectivity over H_2_S and NH_3_; thermally stable	This work
PPyBz-O_TFSI_	269.10	High	High		
PPyBz-O_Br_	271.17	High	High		
PPyTri-O_Cl_	277.38	High	High		
PPyBz-O_PF6_	280.49	High	High		
PPyTri-O_PF6_	284.63	High	High		

## 4. Conclusions

An efficient quaternization of the designed 4,4′-dipyridyl derivative 4,4′-bp-O with the appropriate 1,3,5-tris(bromomethyl)benzene BB and/or cyanuric chloride CC resulted in the effective synthesis of a new class of porous ionic polymers tethering triazine (benzene) nucleus conjugated to three pyridiniums as cationic counterparts tethered bromide/chloride anions PPyBz-O and PPyTri-O. LiTFSI and KPF_6_ anions were incorporated into the core of such polymers through a metathesis protocol furnished on the formation of new ionic polymers. The structures of the formed polymers were investigated by FTIR, ^1^H NMR, and ^13^C NMR analyses. The synthesized polymer was analyzed using PXRD, which showed the presence of semi-crystalline structures. The SEM examination demonstrated that micro or nanoparticles were uniformly distributed within the polymer. Furthermore, the TGA and DTA analyses indicated that the synthesized polymers exhibited good thermal stability. The modified porous ionic polymer was tested for CO_2_ gas sensing using quartz crystal nanobalance (QCN), which demonstrated precise, selective, and reproducible measurements.

## Data availability

The authors confirm that all data are included in the manuscript.

## Conflicts of interest

There are no conflicts to declare.

## Supplementary Material

RA-015-D4RA07062C-s001
