# Correction: Room temperature sensing of CO_2_ using *C*3-symmetry pyridinium-based porous ionic polymers with triazine or benzene cores

**DOI:** 10.1039/d6ra90005d

**Published:** 2026-01-19

**Authors:** Maha A. Alshubramy, Khalid A. Alamry, Hajar S. Alorfi, Sameh H. Ismail, Nadjet Rezki, Mohamed Reda Aouad, Salsabeel Al-Sodies, Mahmoud A. Hussein

**Affiliations:** a Chemistry Department, Faculty of Science, King Abdulaziz University Jeddah 21589 Saudi Arabia maha.alshubramy@gmail.com malshubramy@stu.kau.edu.sa maabdo@kau.edu.sa; b Egypt Nanotechnology Center, Cairo University El–Sheikh Zayed, 6th October Giza Egypt; c Department of Chemistry, Taibah University Al-Madina Al-Mounawara Saudi Arabia; d Chemistry Department, Faculty of Science, Assiut University Assiut Egypt mahussein74@yahoo.com

## Abstract

Correction for ‘Room temperature sensing of CO_2_ using *C*3-symmetry pyridinium-based porous ionic polymers with triazine or benzene cores’ by Maha A. Alshubramy *et al.*, *RSC Adv.*, 2025, **15**, 3317–3330, https://doi.org/10.1039/D4RA07062C.

The authors regret that Fig. 1(A) showed an anomaly in the 4,4′-bp trace, which was caused by automatic graphical markers generated by OriginPro software. The figure should have been as shown herein.

**Fig. 1:**
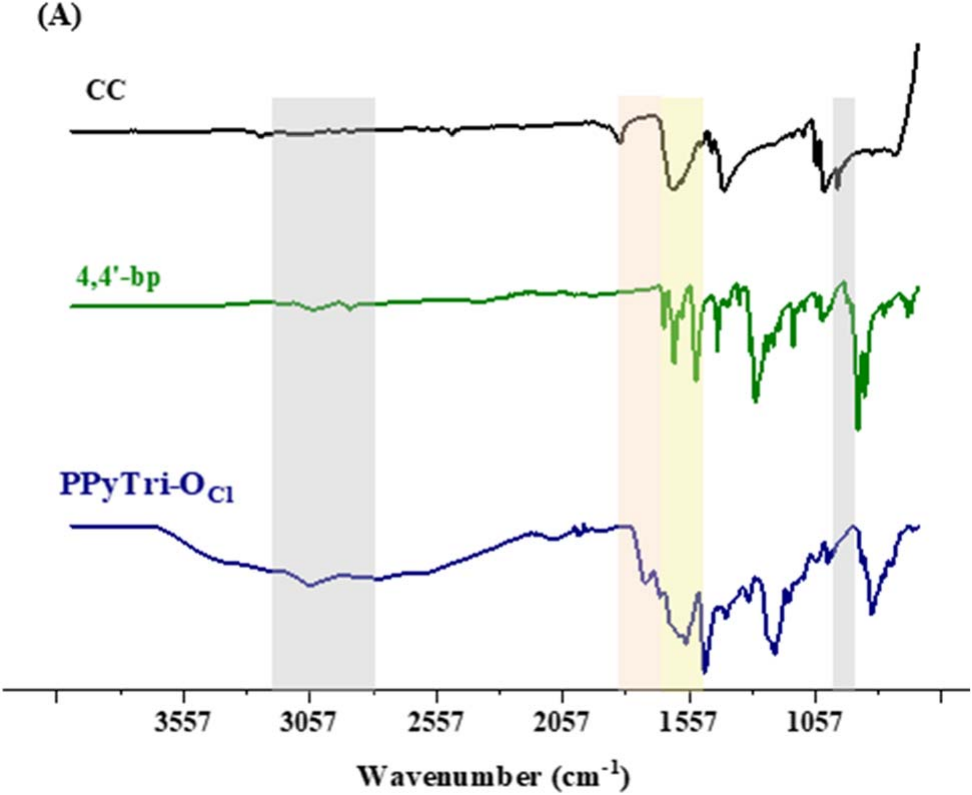
FTIR spectra of the precursor Schiff base 4,4′-bp-O and targeted *C*3-symmetry porous ionic polymers. (A) Triazine core.

The Royal Society of Chemistry apologises for these errors and any consequent inconvenience to authors and readers.

