# Supplementary material for: Room temperature sensing of CO2 using C3-symmetry pyridinium-based porous ionic polymers with triazine or benzene cores
Source: RSC Adv. 2025 Feb 3;15(5):3317–30. doi: 10.1039/d4ra07062c (PMC11788645; doi:10.1039/d4ra07062c)
Supplement: RA-015-D4RA07062C-s001 [file RA-015-D4RA07062C-s001.pdf]

Supplementary Information (SI) for RSC Advances.
This journal is © The Royal Society of Chemistry 2025

Supporting Information File

For

Room-Temperature Sensing of CO_2 Using C3-Symmetry Porous Ionic Polymers Pyridinium-Based with Triazine or Benzene Core

Maha A. Alshubramy,[1]* Khalid A. Alamry,[1] Hajar S. Alorfi [1], Sameh H. Ismail [2], Nadjet Rezki,[3] Mohamed Reda Aouad,[3] Salsabeel Al-Sodies,[1,3] Mahmoud A. Hussein [1,4]*

[1] Chemistry Department, Faculty of Science, King Abdulaziz University, Jeddah 21589, Saudi Arabia.

[2] Egypt Nanotechnology Center, Cairo University, El–Sheikh Zayed, 6th October, Giza, Egypt

[3] Department of Chemistry, Taibah University, 30002, Al-Madina Al-Mounawara, Saudi Arabia

[4] Chemistry Department, Faculty of Science, Assiut University, Assiut, 71516 Egypt

* Corresponding authors' e-mails: (maha.alshubramy@gmail.com), malshubramy@stu.kau.edu.sa; M.A. Hussein (maabdo@kau.edu.sa, mahussein74@yahoo.com).

Contents

No	Item	Title	Page
1	***Chemicals and methods***		**S3**
2	**Figure S1**	FTIR spectra of polymers of PPyBz-O $_{PF6}$, PPyBz-O$_{TFSI}$, PPyTri-O $_{PF6}$, and PPyTri-O$_{TFSI}$	**S4**
3	**Figure S2**	^{19}F NMR and ^{31}P NMR spectra of the polymers PPyBz-O$_{PF6}$, PPyBz-O$_{TFSI}$, PPyTri-O$_{PF6}$ and PPyTri-O$_{TFSI}$	**S5**
4	**Figure S3**	TGA and DTG patterns of synthesized C3-symmetry ionic polymers under air.	**S6**
5	**Figure S4**	Nitrogen adsorption–desorption isotherms and BJH pore size distribution of synthesized C3-symmetry porous ionic polymers	**S7**
6	**Figure S5**	Sensorgram of PIPs responses toward NH_3 or H_2S, showing selectivity for CO_2 gas.	**S8**
7	**Table S1**	Reproducibility Data - Average Δf and RSD (%) of three PIPs sensors at different temperatures	**S9**

1.1 Chemicals and methods

All solvents and chemicals were analytical grades and purchased from Sigma Aldrich. Spectroscopic analysis was used to characterize the produced ionic polymers. At room temperature, ^{1}H NMR (850 MHz) and ^{13}C NMR (200 MHz), ^{31}P NMR (200 MHz), and ^{19}F NMR (200 MHz) spectra were obtained using DMSO-d_6 as solvent. Chemical changes (δ) were measured in parts per million (ppm) using a scale calibrated to the internal standard, tetramethylsilane (TMS). Fourier transform infrared (FT-IR) spectra were recorded using a PerkinElmer Spectrum 100 FT-IR device in the range of 4000-500 cm^{-1}. The morphologies and elemental distributions of the polymers were examined using scanning electron microscopy (SEM, TESCAN VEGA 3, Czech Republic). Samples were mounted on aluminum microscopy stubs using carbon tape and then coated with gold (Au) for 120 s using Quorum techniques Ltd, sputter coater (Q150t, UK). X-ray diffraction (XRD) data were collected using a Bruker D8 Advance X-ray diffractometer operating at 40 kV and 25 mA with a Cu Kα irradiation source. The range of 5°-80° was scanned at a rate of 1°/min. Thermogravimetric analysis (TGA) was performed on a Shimadzu TGA 50 instrument at a heating rate of 10°C/min in air. The Autosorb-iQ surface area analyzer (Quantachrome Instruments Japan GK, Kanagawa, Japan) was used to test the surface area, pore volume, and pore size of the materials at a liquid nitrogen temperature (77 K). To eliminate any moisture or volatiles present within the existing pores of the materials, the samples were preheated at 90°C for 24 hours under vacuum before the measurement. QCN resonators (10 MHz) with gold electrodes (OpenQCN, Italy) were used.

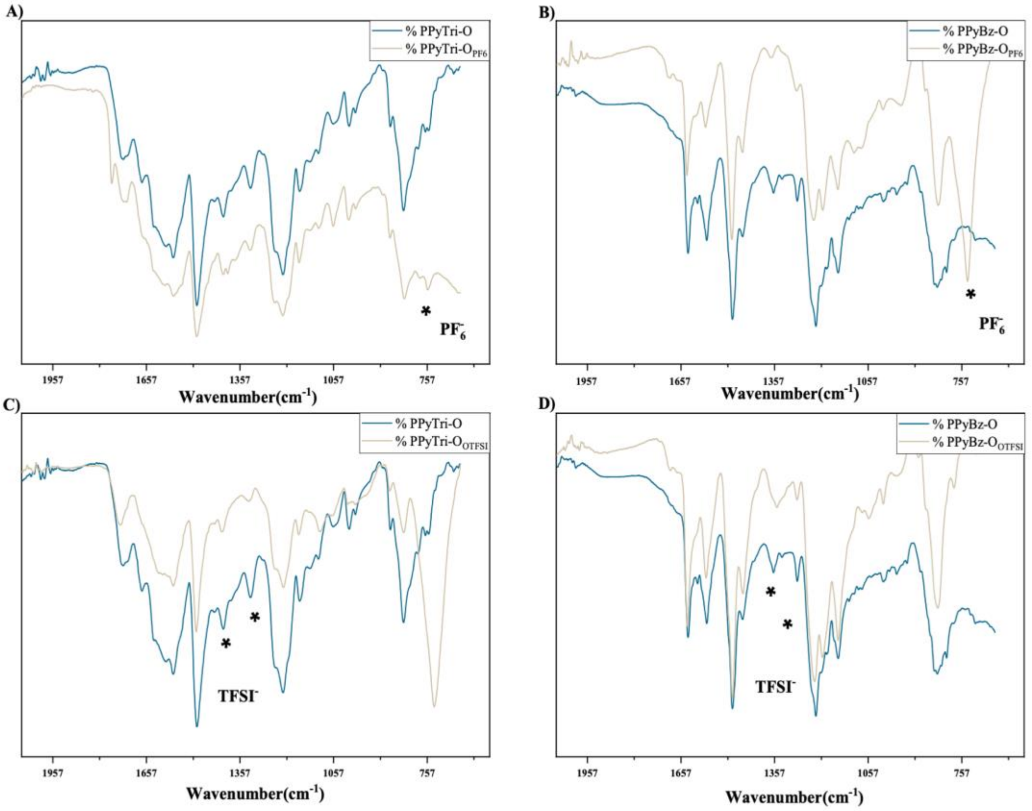

A)
% PPyTri-O
% PPyTri-O$_{PF6}$
*
PF_6^-
1957
1657
1357
1057
757
Wavenumber(cm^{-1})
B)
% PPyBz-O
% PPyBz-O$_{PF6}$
*
PF_6^-
1957
1657
1357
1057
757
Wavenumber(cm^{-1})
C)
% PPyTri-O
% PPyTri-O$_{OTFSI}$
*
*
$TFSI^-$
1957
1657
1357
1057
757
Wavenumber(cm^{-1})
D)
% PPyBz-O
% PPyBz-O$_{OTFSI}$
*
*
$TFSI^-$
1957
1657
1357
1057
757
Wavenumber(cm^{-1})

Figure S1

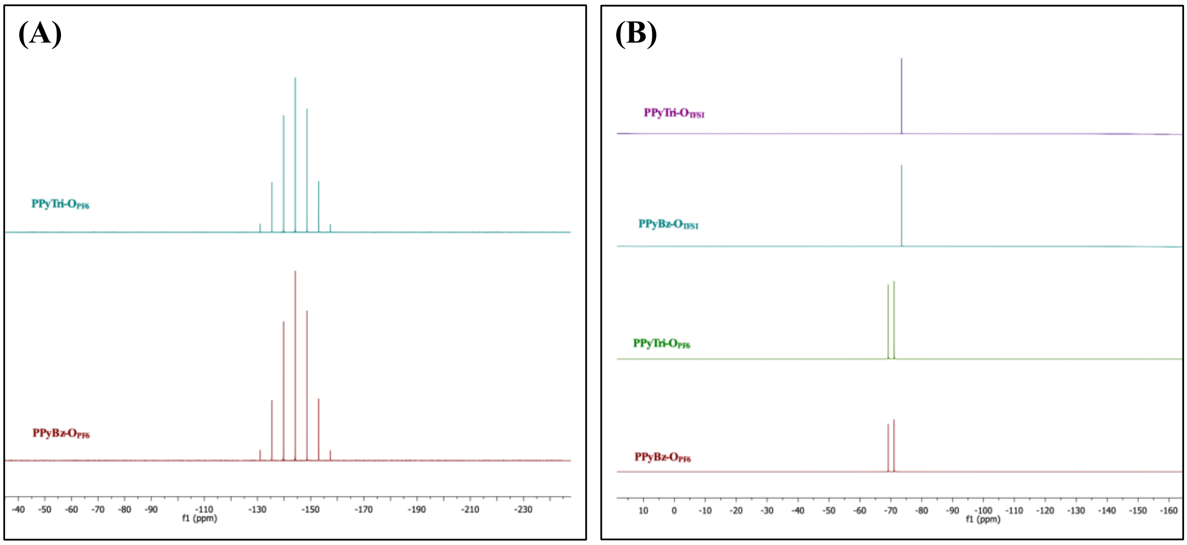
(A)
PPyTri-O$_{PF6}$
PPyBz-O$_{PF6}$
-40 -50 -60 -70 -80 -90 -110 -130 -150 -170 -190 -210 -230
f1 (ppm)
(B)
PPyTri-O$_{TFSI}$
PPyBz-O$_{TFSI}$
PPyTri-O$_{PF6}$
PPyBz-O$_{PF6}$
10 0 -10 -20 -30 -40 -50 -60 -70 -80 -90 -100 -110 -120 -130 -140 -150 -160
f1 (ppm)

Figure S2

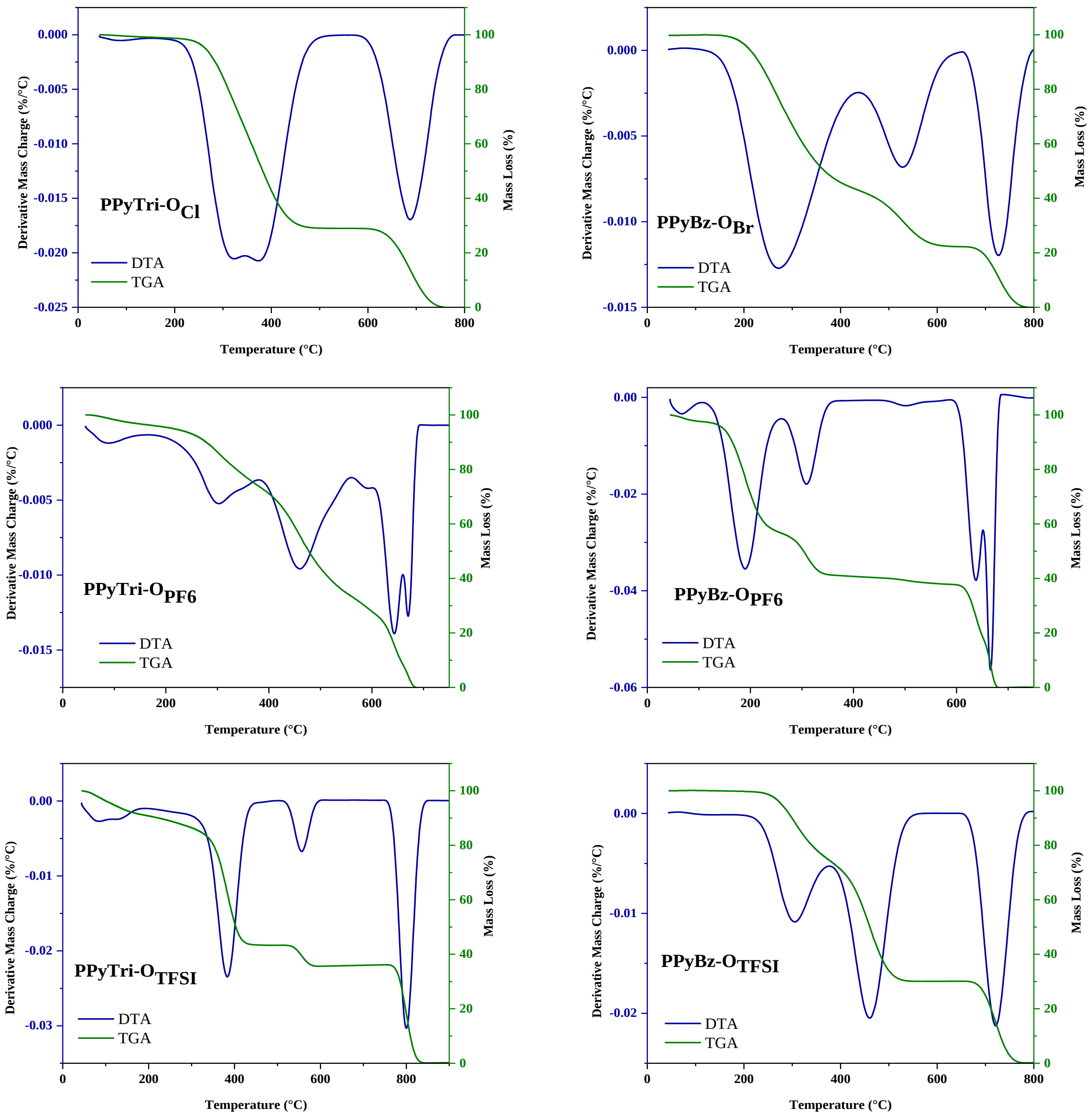
PPyTri-O$_{Cl}$
DTA
TGA
Derivative Mass Charge (%/°C)
Mass Loss (%)
Temperature (°C)
PPyBz-O$_{Br}$
DTA
TGA
Derivative Mass Charge (%/°C)
Mass Loss (%)
Temperature (°C)
PPyTri-O$_{PF6}$
DTA
TGA
Derivative Mass Charge (%/°C)
Mass Loss (%)
Temperature (°C)
PPyBz-O$_{PF6}$
DTA
TGA
Derivative Mass Charge (%/°C)
Mass Loss (%)
Temperature (°C)
PPyTri-O$_{TFSI}$
DTA
TGA
Derivative Mass Charge (%/°C)
Mass Loss (%)
Temperature (°C)
PPyBz-O$_{TFSI}$
DTA
TGA
Derivative Mass Charge (%/°C)
Mass Loss (%)
Temperature (°C)

Figure S3

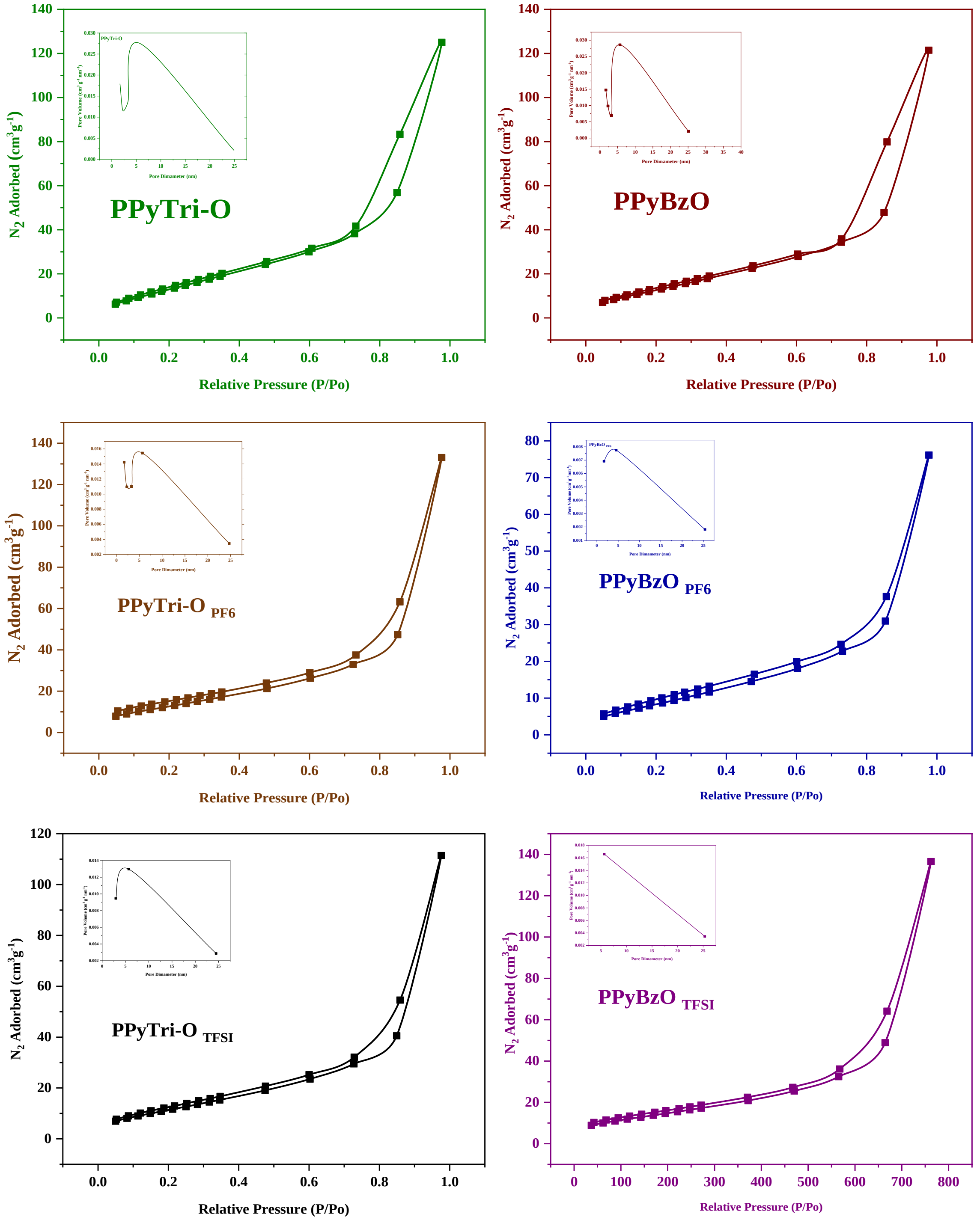

PPyTri-O
PPyBzO
PPyTri-O PF6
PPyBzO PF6
PPyTri-O TFSI
PPyBzO TFSI
N2 Adorbed (cm3g-1)
Relative Pressure (P/Po)
Pore Volume (cm3g-1nm-1)
Pore Dinameter (nm)

Figure S4

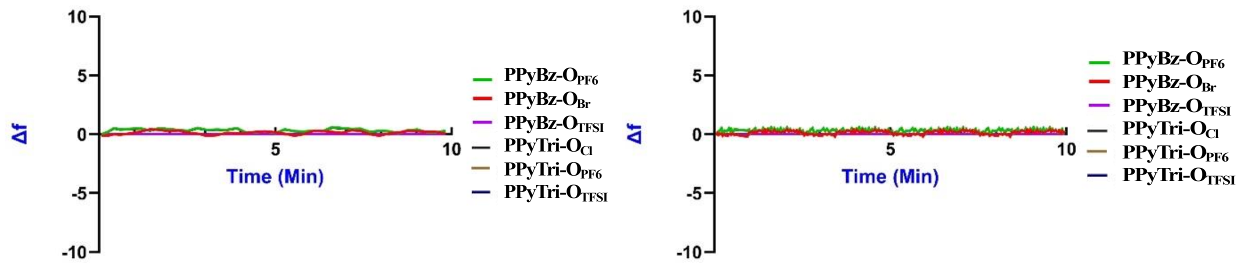

10
5
0
-5
-10
Δf
5
10
Time (Min)
PPyBz-O$_{PF6}$
PPyBz-O$_{Br}$
PPyBz-O$_{TFSI}$
PPyTri-O$_{Cl}$
PPyTri-O$_{PF6}$
PPyTri-O$_{TFSI}$
10
5
0
-5
-10
Δf
5
10
Time (Min)
PPyBz-O$_{PF6}$
PPyBz-O$_{Br}$
PPyBz-O$_{TFSI}$
PPyTri-O$_{Cl}$
PPyTri-O$_{PF6}$
PPyTri-O$_{TFSI}$

Figure S5

Table S.1

Run	Avg. Δf (Hz)			RSD (%)		
	20°C	25°C	30°C	20°C	25°C	30°C
1	253	260	265	1.23	1.47	1.14
2	304	323	332	1.41	1.25	1.34
3	278	285	289	1.75	1.51	1.52
